# Pathophysiology and Perioperative Considerations for Heart Failure Patients

**DOI:** 10.3390/life16081253

**Published:** 2026-07-29

**Authors:** Samuel Crowley, George-Abraam Tawfik, Richard Villa, Claire Larson, Isaac Yeung, Sergio D. Bergese

**Affiliations:** 1 Department of Anesthesiology, School of Medicine, Stony Brook University, 101 Nicolls Rd., Stony Brook, NY 11794, USA; samuel.crowley@stonybrookmedicine.edu (S.C.);; 2 Renaissance School of Medicine, Stony Brook University, 100 Nicolls Rd., Stony Brook, NY 11794, USA; 3 Department of Anesthesiology and Neurological Surgery, School of Medicine, Stony Brook University, 101 Nicolls Rd., Stony Brook, NY 11794, USA

**Keywords:** heart failure pathophysiology, neurohormonal activation, perioperative risk stratification, anesthetic pharmacology, vasopressors, inotropes, invasive hemodynamic monitoring, postoperative fluid management

## Abstract

Patients with heart failure require special considerations in the perioperative setting given the challenges caused by heart failure pathophysiology. This pathophysiology is complex and involves several mechanisms that contribute to both disease progression and the clinical syndrome, including cardiac remodeling, neurohormonal activation, and resultant hemodynamic derangements. Anesthetic management of these patients requires diligence at all stages of care. Preoperative assessment includes risk stratification, management of comorbid conditions, and heart failure medication management. Anesthetic agents have differing effects on hemodynamics and selection of these agents must be aimed at preventing hemodynamic collapse in patients with heart failure. In addition, the increased risk of intraoperative complications among these patients often necessitates means of invasive hemodynamic monitoring and use of vasopressor or inotropic support. Finally, the postoperative care of heart failure patients requires attention to the volume status of these patients, as well as considerations for pain management and modulation of the surgical stress response. In this review article, we aim to review how the pathophysiologic mechanisms and changes that occur with heart failure impact anesthetic management and patient-centered perioperative care.

## 1. Introduction

Heart failure (HF) is a clinical syndrome characterized by structural or functional cardiac abnormalities that results in reduced cardiac output and elevated intracardiac pressures [1]. As of 2023, there are an estimated 64 million people living with HF across the world, with prevalence expected to increase over time [2]. Patients with HF represent a unique challenge for anesthesia providers, as the pathophysiologic changes and symptoms associated with HF can be exacerbated by anesthetic interventions. As such, HF is a significant risk factor for major cardiac adverse events and postoperative complications in patients undergoing surgery [3,4]. This review aims to provide a review of HF pathophysiology and describe the ways this can impact the perioperative anesthetic considerations for these patients, from preoperative risk stratification through postoperative management.

## 2. Methods

An in-depth literature review was conducted for this paper using online databases, including PubMed and Google Scholar. Published peer-reviewed articles focusing on the basic pathophysiology of heart failure, preoperative optimization, intraoperative considerations, and postoperative care of heart failure patients were evaluated. Terms searched for included “Anesthesia”, “Heart Failure”, “Fluid Management”, “Cardiac hypertrophy”, “Neurohormonal Activation”, “Calcium Dysfunction”, “Invasive monitoring”, “Inotropes”, and “Vasopressors.” The date range of all studies included was from 1981 to 2026, with greater emphasis placed on contemporary studies especially in relation to practice guidelines and management recommendations. Key guidelines and studies selected involved multi-institution, evidence-based consensus regarding current practice recommendations. References from included studies were searched manually for further evaluation.

## 3. Pathophysiology of Heart Failure

### 3.1. Cardiac Hypertrophy and Fibrosis

The heart can remodel in response to environmental demands and stimuli. This cardiac plasticity may be physiologic in the case of postnatal growth, adaptation to cardiovascular exercise, or in pregnancy; however, biomechanical stress and pathologic stimuli can trigger pathologic hypertrophy [5]. Hypertrophic growth is a mechanism by which the heart reduces stress on the ventricular wall in accordance with the Law of Laplace [6,7,8]. Persistent pressure overload, such as in hypertension, valve disease, or after infarct and neurohormonal factors, results in paracrine and autocrine signaling, which stimulates hypertrophic growth through a complex network of signaling molecules and intracellular cascades [9]. Insulin-like growth factor (IGF) I, angiotensin II, and endothelin-1 have been demonstrated to play a role in vitro [10] and in patients with aortic stenosis [11]. The resultant hypertrophy plays a role in the development of HF as it eventually progresses to cardiac dilation and impaired contractility.

In addition to hypertrophy, it is now known that myocardial interstitial fibrosis plays an important role in the development of HF [12]. Cardiomyocyte death or injury stimulates cells to produce fibrous tissue and secrete profibrotic mediators [13]. This ultimately leads to changes in the extracellular matrix, which results in an excess of collagen type I and II fibers, and an environment that favors fiber deposition versus degradation [14]. The accumulation of fibrotic tissue leads to left ventricular dysfunction and can contribute to arrhythmia and decreased myocardial oxygen availability [12].

### 3.2. Neurohormonal Activation

The damage to cardiac tissue that occurs from acute injury or develops over time, as with persistent pressure overload, has the ultimate hemodynamic consequence of reduced cardiac pump function. This, in turn, results in a decreased “effective” arterial circulating volume, which is sensed by peripheral arterial baroreceptors and chemoreceptors, activating a series of compensatory mechanisms to maintain cardiac hemostasis [15]. These compensatory mechanisms include activation of the sympathetic nervous system (SNS), the renin–angiotensin–aldosterone system (RAAS), and through inflammatory mediators involved in repair of cardiac injury [15,16]. Persistent activation of these compensatory mechanisms eventually becomes maladaptive, leading to HF. This concept was formalized by Packer in 1992, who noted that an understanding of hemodynamic derangements explains the symptoms of HF, but not the progression of the disease process. His “neurohormonal hypothesis” states that an overexpression of biologically active molecules drives disease progression through the deleterious effects of sustained neurohormonal activation on the heart and circulation [17].

Decreased effective circulating volume is sensed by baroreceptors in the aorta and carotid sinus, which leads to activation of the SNS and a decrease in activity of the parasympathetic nervous system. This leads to an increased heart rate and contractility, as well as peripheral vasoconstriction. Over time, sustained activation of the SNS leads to increased circulation levels of adrenergic neurotransmitters in the plasma, as well as an imbalance between sympathetic and parasympathetic tone, which are implicated in progression of HF [18]. At the level of the kidney, increased SNS activity, as well as the decreased effective circulating volumes, lead to activation of the RAAS, as the body aims to increase cardiac output by increasing salt and water retention and maintain blood pressure through vasoconstriction [15]. The release of renin prompted by beta-1 adrenergic stimulation [19], as well as decreased blood flow in the juxtaglomerular apparatus, converts angiotensinogen into angiotensin I [20]. Angiotensin I is converted to angiotensin II (ATII) by angiotensin converting enzyme (ACE). ATII is a potent vasoconstrictor and induces the release of aldosterone from the zona glomerulosa of the adrenal glands, leading to sodium and water retention [20]. In HF patients, there is persistent activation of the RAAS and resistance to mechanisms which oppose this system, leading to progression of disease and volume overloaded states. Atrial natriuretic peptides and brain natriuretic peptides are produced in response to atrial or myocardial stretch, which cause vasodilation and renal sodium and water excretion [21]. Normally an important countermeasure to the RAAS, in HF there is resistance to natriuretic peptides, leading to unopposed vasoconstriction and edema [21,22,23]. In addition to resistance to these peptides, there is also evidence for continued production of aldosterone [24] and desensitization to beta-adrenergic stimulation [25]. These mechanisms represent some of the changes to regulatory neurohormonal systems resulting in the progression of HF and its symptoms.

Over time, persistent abnormal neurohormonal activation also contributes to cardiac remodeling, as described above, and to contractile dysfunction, both of which worsen pump function. As mentioned, there is desensitization to adrenergic stimulation, with decreased beta-receptor mediated contractile responses [26] and decreased expression of beta-1 receptors [27]. Another contributor to contractile dysfunction seen in HF is abnormal calcium metabolism. Excitation–contraction coupling entails the release of Ca^2+^ from the sarcoplasmic reticulum and the binding of these ions to myofilaments to initiate contraction [28]. Ca^2+^ is then taken back into the sarcoplasmic reticulum via a Ca^2+^-ATPase pump (SERCA2a) and cellular efflux via the sodium–calcium exchanger. In HF, there is leak from SERCA2a and other receptor ion channels, which leads to decreased Ca^2+^ amplitude, which in turn contributes to a decreased contractile force [29]. Changes in cellular calcium and sodium regulation also contribute to the increased risk for arrhythmia seen in HF [30]. Risk of arrhythmia is also influenced by changes to cardiac geometry, contractile tissue composition, and electrophysiological remodeling [31].

### 3.3. Hemodynamic Consequences of Heart Failure

The ultimate effect of these pathophysiologic mechanisms is an inability of the heart to provide adequate cardiac output without incurring increased cardiac filling pressures [32]. In a normal cardiac cycle, ventricular and atrial pressures increase to propel blood forward during systole, while during diastole there is relaxation to allow for filling of the cardiac chambers. As a chamber fills, increased volume is related to increased stretch of myocardial elements, which results in increased pressure. In a normally functioning heart, an increase in preload, commonly measured as left ventricular end-diastolic pressure (LVEDP), results in an increase in systolic function [33]. The pressure–volume relationship is also influenced by the contractile ability of the myocardial tissue and the external restraint to forward flow on the heart or afterload [32]. In HF, there is a spectrum of impaired forward flow, which ranges from decreased cardiac output reserve during exercise to the extremes of cardiogenic shock, where cardiac output is insufficient to preserve proper organ function at rest [34]. This impaired forward flow results in elevated cardiac filling pressures, which is worsened by volume retention mediated by the neurohormonal axis and leads to organ dysfunction in the lungs and kidneys. On the left side of the heart, congestion leads to elevated pulmonary artery wedge pressures (PAWPs), increasing hydrostatic pressure in the pulmonary vasculature and leading to pulmonary edema [34]. Because the left and right side of the heart are connected in series, elevated filling pressures on the left side of the heart will also lead to elevated right-sided pressures and increased central venous pressure (CVP) [35]. As such, with progression of HF, venous congestion is also seen with end-organ dysfunction in the kidneys [36], as well as peripheral edema, particularly in decompensated states.

## 4. Preoperative Assessment

HF increases perioperative morbidity and mortality due to impaired cardiac reserve, chronic neurohormonal activation, and increased vulnerability to the hemodynamic stress associated with anesthesia and surgery [37]. The body’s physiologic response to surgical stress involves dynamic changes in preload, afterload, heart rate, and contractility [37]. HF patients do not have the cardiac reserve to adapt to these changes, making them susceptible to hypotension, fluid overload, arrhythmias, and cardiac decompensation in the perioperative period. As a result of the risk for patients with HF undergoing surgery, it is essential to identify and optimize each patient prior to surgery. The 2024 American Heart Association/American College of Cardiology (AHA/ACC) Guidelines for Perioperative Cardiovascular Management for Noncardiac Surgery emphasizes a multidisciplinary, team-based approach to the management of complex conditions in the perioperative setting [38]. Patients with HF fit into this category, and interdisciplinary models may be of benefit to standardize perioperative cardiac care practices and accelerate coordination of recovery activities [38].

### 4.1. Risk Stratification

The diagnosis of HF is classified in different ways. One common classification is based on left ventricular ejection fraction (LVEF): heart failure with reduced ejection fraction (HFrEF), LVEF < 40%; mildly reduced ejection fraction (HFmrEF), LVEF 41–49%; and preserved ejection fraction (HFpEF), LVEF ≥ 50%, but with diastolic dysfunction [39]. Another widely used system is the New York Heart Association (NYHA) functional classification, which categorizes patients based on symptom severity: Class I, no limitation in physical activity, to Class IV, symptoms at rest, with increasing levels of limitations in between [40]. In addition to the two classification systems already mentioned, the American College of Cardiology/American Heart Association (ACC/AHA) staging system describes disease progression from Stage A (at risk) to Stage D (advanced heart failure).

Patients with advanced heart failure like NYHA class III or IV or ACC/AHA Stage C or D disease carry the highest risk in the perioperative setting [40]. One of the objective measures used to assess systolic function is the ejection fraction. An LVEF < 30% indicates severe systolic dysfunction, which massively limits the ability to tolerate surgical stress [39].

Metabolic equivalents (METs) are another commonly used metric to identify perioperative risk and likelihood of complications. METs are estimated by asking patients about their physical activities and correlating those activities with MET values using standardized activity scales. It is well known that patients with low METs are at increased risk for perioperative morbidity and mortality [41]. Additional perioperative risk indices such as the Revised Cardiac Risk Index (RCRI), the Surgical Outcome Risk Tool, and the 21-component universal National Surgical Quality Improvement Program (NSQIP) may also be used to characterize surgical risk of patients with cardiac or other conditions, including HF [38]. Additionally, recent hospitalization, worsening symptoms of heart failure, and elevated biomarkers such as B-type natriuretic peptide (BNP) or N-terminal pro–BNP (NT-proBNP) increase perioperative morbidity and mortality [42].

HF is a complex and dynamic pathologic process, and patients with advanced HF may become clinically decompensated or hemodynamically unstable in the course of their disease. In these circumstances, the 2024 AHA/ACC guidelines says that consideration should be given to postponing elective surgery, and further work to optimize patients, such as cardiology consultation, may be beneficial to assist with perioperative management [38].

### 4.2. Comorbidity Management

HF rarely exists in isolation and is frequently seen with additional comorbidities, which is why a comprehensive perioperative evaluation should be considered in these patients. As previously discussed, these different organ systems work in conjunction with one another. As such, the development of HF often parallels the development of comorbid conditions.

Renal dysfunction is common in patients with HF and may impair the ability to regulate fluid and electrolyte balance. Acute fluid shifts in patients with renal dysfunction can precipitate worsening renal injury or exacerbate HF symptoms. Optimizing renal function by correcting electrolyte derangements before surgery helps reduce negative outcomes [43].

Pulmonary disease and pulmonary hypertension can further complicate perioperative management by increasing right ventricular afterload and impairing oxygenation. In addition to an increase in afterload, hypoxia and hypercapnia may worsen pulmonary vasoconstriction, increasing the risk of right ventricular failure [44,45].

Diabetes mellitus should be taken into consideration, since poorly controlled disease increases the risk of ischemic events due to microvascular damage. Anemia should be screened for and optimized in patients with HF, as poorly controlled anemia reduces oxygen delivery to tissues and may worsen myocardial ischemia during physiologic stress [44,46].

### 4.3. Heart Failure Medication Management

Guideline-directed medical therapy (GDMT) forms the foundation of HF management and slows the progression of HF. GDMT for patients with HFrEF consists of four major medication classes: beta-blockers, renin–angiotensin system inhibitors, mineralocorticoid receptor antagonists, and sodium glucose cotransporter-2 (SGLT2) inhibitors [47].

Several studies have suggested continuing β-blockers during the perioperative period in patients receiving chronic therapy, as abrupt cessation increases cardiovascular morbidity and mortality [48]. Maintaining heart rate control is important in HF patients to preserve cardiac output [49]. Abrupt withdrawal of β-blockers can lead to rebound tachycardia and increased myocardial oxygen demand [50].

In recent years there has been much discussion about the use of angiotensin converting enzyme inhibitors (ACEIs) and angiotensin receptor blockers (ARBs) in the perioperative period due to profound intraoperative hypotension during the administration of anesthesia, which is often poorly responsive to fluid resuscitation and conventional vasopressors. Recommendations regarding the perioperative use of these medications are continually evolving, with the most recent guidelines from the AHA/ACC in 2024 stating that omission 24 h before surgery may be beneficial to limit intraoperative hypotension [38]. However, the guidelines draw a distinction between patients taking these medications for hypertension and note that in patients on chronic renin–angiotensin–aldosterone system inhibitors for HFrEF, perioperative continuation is reasonable, adding that HF medication regimens should ideally have minimal interruption [38].

Diuretics require careful consideration as well and should be decided on a case-by-case basis. In patients who show signs of acute congestion, continuation may be necessary to prevent fluid overload. However, excessive diuresis may result in hypovolemia and perioperative hypotension [51]. In addition to hypotension, diuretics including loop diuretics have the risk of electrolyte derangements and could ultimately provoke arrhythmias [52].

SGLT2 inhibitors have shown benefits in heart failure management but may require temporary discontinuation prior to surgery. Euglycemic ketoacidosis is a rare complication of SGLT2 inhibitors that can be seen in the postoperative period. The specific risk of this complication in patients with HF is unclear; however, the 2024 AHA/ACC guidelines recommend these agents be stopped 3 to 4 days before elective surgery [38]. There are no consensus recommendations regarding resumption of these medications; however, patients should be hemodynamically stable and have resumed a normal diet before resuming.

Overall, the most recent guidelines from the AHA/ACC quote it is “reasonable to continue GDMT (excluding SGLT2 inhibitors) in the perioperative period, unless contraindicated, to reduce the risk of worsening HF” [38].

## 5. Anesthesia Considerations

When caring for patients with HF, it is critical to understand how the pathophysiology of HF affects perioperative management and predisposes patients to a higher risk of perioperative hemodynamic instability. It is important to be aware of higher-risk periods for alterations in hemodynamics during the surgical period, and ensure the maintenance of adequate preload and avoid significant periods of hypotension. Perioperative hypotension may arise from the direct effect of anesthetics, side-effects of other intraoperative medications administered, mechanical ventilation, intraoperative fluid shifts, surgical insufflation, and manipulation of the heart and major vessels during intrathoracic surgery. It is crucial for anesthesiologists to proactively predict such periods of hypotension and ensure the maintenance of hemodynamic stability.

### 5.1. Perioperative Hemodynamic Management

During the perioperative period, surgical/anesthetic stressors can exacerbate HF. Cardiac dilation, impaired contractility with increased risk for systemic and pulmonary edema, accumulation of cardiac fibrotic, arrhythmias, and decreased myocardial oxygen availability all make perioperative hemodynamic management increasingly challenging. Patients with HF need adequate preload but cannot tolerate excessive volume. Decreased preload results in decreased stroke volume, cardiac output, and ultimately hypotension. On the other hand, excessive volume results in increased chamber pressures, pulmonary congestion and edema. As such, goal-directed fluid administration is needed, as both hypovolemia and excessive fluid administration in patients with HF are associated with complications and morbidity [53]. Careful assessment of a patient’s volume status must be made continuously during the administration of anesthesia for HF patients, as fluid administration may be a necessary step in addressing hemodynamic changes; however, in cases where a patient has adequate preload, fluids should be used judiciously and the support of vasopressors or inotropic agents may be needed, as we will discuss. Sympathetic stimulation during surgery can lead to hypertension, tachycardia, impaired left ventricular relaxation, and wide fluctuations in blood pressure, which can cause an imbalance in oxygen supply and demand. Intraoperative hypotension, bleeding, and anemia can also result, which can decrease preload and cardiac output, result in inadequate organ perfusion, and further the imbalance between oxygen supply and demand [54]. Additionally, the development of arrhythmias with an elevated ventricular rate can decrease diastolic filling time and diminish the atrial kick, which is critical for maintaining hemodynamic stability in patients with HF [55]. A deficit of myocardial oxygen supply in relation to demand can result in ischemia and myocardial injury. Patients with HF often have increased ventricular stiffening, elevated left ventricular filling pressures, and reduced arterial compliance, and are significantly sensitive to decreases in preload or afterload, which can occur during surgery [55]. For these reasons, optimization of hemodynamics is crucial in the perioperative period.

### 5.2. Activation of RAAS, SNS and Perioperative Considerations

The over-stimulation of the RAAS and SNS in HF may contribute to perioperative hypotension and challenges with hemodynamic management due to resulting desensitization to beta-adrenergic stimulation. Adrenergic downregulation results in progressive reduction in a cardiovascular response to exogenous catecholamine administration [56]. Patients with HF undergoing surgery have an increased risk of vasoplegia (vasodilatory shock), which is characterized by hypotension and the continued need for vasopressors despite a normal or high cardiac index [57]. The chronic endogenous over-stimulation of beta-adrenergic receptors, resultant downregulation of these receptors, and ultimate increased resistance to vasopressors is one mechanism that could contribute to the development of vasoplegia intraoperatively [57]. Additionally, this desensitization to beta-receptors makes patients more vulnerable to the systemic inflammatory response that may arise from surgical trauma [57]. Furthermore, studies have shown that patients with HF are more vulnerable to developing arrhythmias with sympathetic stimulation [58], and the use of beta-adrenergic agonist treatment is associated with increased mortality in HF patients [59]. Further research is needed to provide more definitive vasopressor guidance perioperatively; however, current studies have shown non-adrenergic vasopressors such as vasopressin have been effective in treating hypotension and maintaining hemodynamic stability perioperatively [60]. In regards to the pathophysiology of HF, possible reasons for this finding are that heart failure patients have adrenergic hyposensitivity and the use of high doses of catecholamines may lead to significant adverse effects such as arrhythmias, organ ischemia, and increased mortality [60]. Similarly, norepinephrine is commonly used and recommended due to its strong alpha-agonist properties and ability to restore systemic perfusion [60]. However, due to norepinephrine’s beta-agonism, treatment with high doses of norepinephrine are associated with tachycardia, tachyarrhythmia, hyperglycemia, and lactic acidosis [61]. The use of medications to support contractile function may still be indicated in the perioperative setting, but an understanding of possible side-effects is important to bear in mind regarding their use.

### 5.3. Interplay Between Calcium Dysfunction in Heart Failure and the Effect of Anesthetics

As mentioned previously, another key mechanism in the pathophysiology of HF is abnormal calcium metabolism, which can decrease cardiac contractile force and exacerbate arrhythmias. This is important in regards to perioperative management, as anesthetics have been found to affect voltage-gated calcium channels (VGCCs), including those involved in cardiac muscle contraction [62]. For example, L-type calcium channels were found to drive cardiac muscle contraction and regulate vascular tone [63]. Anesthetics such as propofol, volatile anesthetics, and local anesthetics were found to inhibit voltage-gated calcium channels, including L-type VGCCs, which could further contribute to their cardio-depressant effects. Parikh et al. found that volatile anesthetics and propofol can further inhibit these channels through the phosphatidylinositol 4,5 bisphosphate (PIP2) pathway. Parikh et al. found that modification of PIP2 signaling by anesthetics can alter VGCC behavior, thus affecting calcium channel functioning beyond simple direct inhibition of calcium channels [64]. Additionally, dysfunction of VGCCs can exacerbate arrhythmias [62], further complicating the management of pre-existing HF and anesthetic administration. Thus, anesthetic inhibition and alteration of calcium channels in conjunction with heart disruption of calcium channel functioning requires greater care and surveillance during the administration of anesthesia to prevent significant hypotension, arrhythmias and hemodynamic instability. The administration of calcium intraoperatively can be considered for transient hemodynamic support, as increased mean arterial pressure (MAP) was shown after administration but effects were found to fade within 10–20 min [65]. Furthermore, Datt et al. found that vasoplegia from the resulting disruption of calcium channels can result in resistance to catecholamine vasopressors that was best treated with vasopressin, norepinephrine, or rescue medications such as hydroxocobalamin and methylene blue [66]. More research is needed to further investigate the long-term effects of anesthetics’ effect on calcium channels and resulting alteration of systemic function.

### 5.4. Induction of General Anesthesia

One period which has an especially high risk for hemodynamic instability is induction. One study by Maheshwari et al. found that about one-third of intraoperative hypotension occurred between induction and the first surgical incision [67]. Bearing in mind that HF patients have poor cardiac reserve and persistent, maladaptive activation of the SNS to increase cardiac output in the setting of pump dysfunction, clinicians should be wary of decreased sympathetic tone that can occur with induction, which may yield hemodynamic collapse. A key point is that a slow, controlled induction is crucial to prevent significant drops in blood pressure and cardiac output, with gradual titration of induction medications and careful assessment of response. Furthermore, the use of pre-induction invasive monitoring, such as an arterial line, can be helpful to provide beat-to-beat blood pressure monitoring and assessment of the hemodynamic response to induction.

In regard to the agents used to induce general anesthesia, a comprehensive understanding of their effects is critical to result in a safe induction. For instance, propofol is widely used for inducing general anesthesia, but can induce hypotension, depress myocardial activity, and decrease systemic vascular resistance [68]; thus, vasopressors should be readily available to address any hypotension that may arise from its vasodilatory effects. Etomidate can be considered for induction due to its ability to cause fewer systemic hemodynamic changes than other induction agents; however, it can suppress the adrenocortical axis, and thus it may not be suitable for critically ill patients [69]. Ketamine can also be considered as an induction agent as it has been shown to maintain systemic vascular resistance (SVR), but it can lead to increased secretions and an increase in intracranial pressure [70]. Furthermore, the maintenance of SVR when using ketamine relies on patients’ endogenous catecholamines and thus may not have the effect of maintaining SVR in patients who are catecholamine depleted. Opioids used intraoperatively aid in reducing the sympathetic response to surgical stimulation; however, they also reduce cortisol secretion, which can contribute to hypotension [54]. Regardless of which induction agent is chosen, the most important aspect is that a slow controlled induction is employed in which medications are titrated slowly and carefully to assess hemodynamic response.

### 5.5. Maintenance of Anesthesia and Anesthetic Technique

Another important perioperative consideration is selecting a method for maintaining anesthesia. Volatile anesthetics are frequently used for the maintenance of general anesthesia; however, volatile anesthetics have been shown to cause reversible myocardial depression and decrease systemic vascular resistance. Some studies have found that the use of volatile anesthetics may have cardioprotective effects compared to total intravenous anesthesia (TIVA) with propofol through mechanisms such as modulation of G-protein coupled receptors, intracellular signaling pathways that result in a decrease in cellular oxygen demand, regulating expression of apoptosis-related genes, and reducing oxidative stress during hypoxia [71]. However, recent multi-center randomized control trials found no significant cardioprotective effects when comparing the difference in troponin elevation, incidence of ischemia, or postoperative major adverse cardiac events between the use of volatile anesthetics vs. TIVA [72,73,74,75]. Thus, there does not appear to be a clear benefit to choosing maintenance of general anesthesia with volatile anesthetics vs. TIVA, and the choice between maintaining anesthesia with volatile anesthetics vs. TIVA is less important than ensuring hemodynamic stability and avoidance of hypotension with whichever technique is used.

As surgical conditions allow, anesthetic technique should also be carefully considered when caring for patients with HF. For example, using regional anesthesia rather than general anesthesia can avoid systemic hypotension resulting from use of general anesthetics and the effects of positive pressure ventilation [70]. Positive pressure can be deleterious to patients with HF through increases in pleural pressure and ultimately decreased right and left ventricular preload [76,77]. For instance, one study by Chahrour et al. compared the use of regional anesthesia (RA) vs. general anesthesia (GA) in patients with congestive heart failure undergoing lower extremity amputation and found that the use of RA was associated with decreased morbidity and mortality compared to GA [78]. When using neuraxial techniques, however, sudden drops in preload and afterload should be prevented and the concurrent use of vasopressors can provide stable hemodynamics while still maintaining favorable surgical conditions [77].

Furthermore, adequately controlling ventilation is especially important for patients with right heart failure or pulmonary hypertension. For instance, hypoventilation and hypercarbia can further increase pulmonary vascular resistance, worsening pulmonary hypertension, and exacerbate right heart failure, as the right ventricle may be unable to pump across the increased pulmonary vascular bed pressure. When caring for patients under regional or neuraxial techniques, care must be taken to avoid excessive sedation due to the precipitation of hypoventilation and hypercarbia. Additionally, when intubation is necessary and positive pressure ventilation is employed, intrathoracic pressure and tidal volumes should be minimized as tolerated and minute ventilation should be controlled to prevent hypoventilation [77].

### 5.6. Role of Invasive Monitoring

Rigorous monitoring of hemodynamics with invasive monitoring may be indicated in the perioperative period. For instance, an arterial line can be placed when beat-to-beat monitoring of blood pressure and/or frequent blood sampling is warranted intraoperatively. During each cardiac contraction, pressure is exerted that results in mechanical motion of flow within the arterial catheter. This mechanical motion gets transmitted to a transducer through a rigid fluid-filled tube. The transducer then produces a beat-to-beat arterial waveform and numerical pressures including systolic, diastolic, and mean arterial pressure [79]. This is important, as the monitoring of beat-to-beat blood pressure allows for tighter blood pressure control than non-invasive periodic cuff readings. This is especially important during periods of expected hemodynamic shifts and fluctuations in blood pressure, such as during induction or critical surgical portions. The use of an arterial line may also be able to assist with determinations of fluid responsiveness through the use of pulse pressure variation (PPV) in anesthetized patients under mechanical ventilation [80]. As mentioned previously, hemodynamic optimization is of paramount importance in HF patients, as inadequate preload may not be tolerated, but excessive fluid administration can predispose to volume overload. However, further research is needed regarding the accuracy of PPV in HF patients, as concerns have been raised that cardiac dysfunction may limit the diagnostic accuracy of PPV [81]. Furthermore, an arterial line allows for frequent lab draws, such as for arterial blood gases, which give clues towards acid–base status and ventilation, electrolyte, and transfusion management.

A central venous catheter with monitoring of CVP may also be indicated when caring for patients with HF perioperatively. The gold standard for measurement of central venous pressure is when the central venous catheter is positioned, as it transitions into the right atrium [82]. CVP and its waveform are generated by transmitted intraluminal pressure viewed over time. This produces a characteristic waveform across the cardiac cycle. CVP can be used to estimate right atrial pressure and be used as a surrogate for right ventricular diastolic pressure [83]. Changes in the CVP waveform can be used to analyze fluid status, tricuspid valvulopathy, and right ventricular systolic and diastolic function [83].

Additionally, the use of a pulmonary artery catheter may provide information such as pulmonary artery pressure, cardiac output calculation, and mixed venous oxygen saturation. This information can help guide intraoperative fluid administration and vasoactive therapies. However, the placement of a pulmonary artery catheter is not without risks. Some risks include bleeding, infection, inadvertent arterial puncture, dysrhythmias, and pulmonary artery injury. Thus, the American Society of Anesthesiologists (ASA) taskforce does not recommend routine use of pulmonary artery catheterization in low-risk patients, but is appropriate for select surgical patients, including those undergoing procedures with expected significant hemodynamic changes, such as cardiac surgery or those with significant pre-existing risk factors such as advanced cardiopulmonary disease [84]. As perioperative fluid management is especially challenging in patients with HF, invasive monitoring, such as via central venous catheters or pulmonary artery catheters, can provide additional data to aid in guiding fluid management.

Intraoperative transesophageal echocardiography (TEE) can also be utilized to help guide management. Some indications for perioperative TEE include surgery for high-risk patients such as those with significantly reduced ejection fraction, decompensated HF, expected intraoperative hemodynamic instability, or expected major fluid shifts during the surgical procedure. However, there are contraindications for TEE, which include patient refusal, post-esophageal surgery, esophageal pathology such as tumors, esophageal tears, or tracheoesophageal fistulas. Performing TEE intraoperatively can provide information regarding ventricular structure and function, cardiac output, valvular function, and volume status, and guide intraoperative management in real time [85].

### 5.7. Pharmacologic Management with Vasopressors and Inotropes Perioperatively

When managing HF perioperatively, inotropes and vasopressors can be utilized based on clinical need. Several meta-analyses have shown the equivalence or superiority of non-adrenergic vasoconstrictor medications in regards to reductions in morbidity and mortality compared to catecholamines in vasodilatory states [86,87]. When used for resuscitation, vasopressors are primarily used when the main clinical benefit end goal is to raise MAP to rapidly restore organ perfusion pressure by correcting the deficit in SVR [88]. When selecting for inotropes, the main clinical benefit end goal is to increase contractility to increase stroke volume and cardiac output to restore adequate oxygen delivery to tissues [88]. However, the use of inotropes in high doses may lead to adverse effects such as arrhythmias, organ ischemia, and increased mortality [89]. It must be noted that some vasopressors have inotropic properties, and some inotropes have vasopressor properties as well. In HF patients, careful consideration must be given to the suspected cause of hemodynamic instability, and an understanding of the pathophysiology of heart failure can help guide clinical decision making.

Under anesthesia, vasodilation remains the most common cause of hypotension and, as such, vasopressors are typical first-line agents in surgical patients [79]. The pure alpha-agonist phenylephrine is frequently used to maintain MAP via increased SVR and venous return, which can augment preload [79]. Phenylephrine will also cause alpha-1 activation in lung vasculature and can increase pulmonary vascular resistance, which may make this agent less suitable for patients with pulmonary hypertension [88]. Vasopressin may also be employed and acts via the V1 receptor to act as a potent vasoconstrictor without increasing pulmonary vascular resistance, which may provide an additional benefit in patients with right heart failure and pulmonary hypertension [70]. In patients with heart failure, vasoplegia under anesthesia is a common cause of hypotension and utilization of vasopressor agents is reasonable. However, by increasing SVR, these agents will increase afterload and may be poorly tolerated by patients with pre-existing pump dysfunction and may decrease cardiac output [88]. In these scenarios, it may be more appropriate to select agents with inotropic activity so that the contractility of the heart is supported to improve forward flow.

The beta-1 agonist dobutamine can be used to increase contractility and cardiac output; however, tachycardia may occur, which can decrease diastolic filling time [90]. Norepinephrine is often used perioperatively and mainly exerts its effects through potent alpha-1 activity with additional beta-1 agonism to increase myocardial contractility, heart rate, and peripheral vascular resistance to maintain coronary perfusion pressure [91]. In heart failure patients under anesthesia, norepinephrine may be of particular interest, as potent alpha-1 activity can increase SVR and counter vasodilation caused by anesthetics, while modest beta-1 activity can support contractility. Increases in heart rate and cardiac output are less than with epinephrine, as alpha-activity predominates; so, in cases of severe systolic dysfunction, caution must be exercised, as the increase in afterload can result in a decreased cardiac output, as with vasopressors [88,92]. Epinephrine has potent agonism of alpha and beta receptors, which can both increase contractility and peripheral vascular resistance; however, it can increase myocardial oxygen demand [93] and has an increased potential to exacerbate arrhythmias. At lower doses, it results in increased cardiac output via predominantly beta-1 agonism with decreased SVR and variable effects on MAP. At higher doses, alpha-1 vasoconstrictive effects are dominant, which produce increased systemic vascular resistance, MAP, and cardiac output [92].

HF patients may also present with signs of malperfusion or end-organ dysfunction despite adequate blood pressures. In these scenarios, support of contractility as well as augmentation of forward flow through reduced afterload can be achieved with the use of inodilators. Phosphodiesterase inhibitors such as Milrinone have direct inotropic and lusitropic (cardiac relaxation) effects by increasing cyclic adenosine monophosphate (C-AMP) [94]. Additionally, they have indirect positive effects on cardiac function by reducing afterload and can also lead to decreases in pulmonary vascular resistance. An additional benefit of phosphodiesterase inhibitors is that they have a reduced risk of inducing arrhythmias compared to dobutamine or epinephrine [94]. However, phosphodiesterase inhibitors may lead to hypotension, so they must be used cautiously and often with the supplementation of vasopressors [91]. The calcium sensitizer Levosimendan has inotropic and vasodilatory effects, and studies have shown that it exhibits short-term efficacy in right heart failure, but caution must be taken to avoid systemic hypotension [95]. A randomized control trial by Shaker et al. found that patients with heart failure and an EF < 35% who received a Levosimendan infusion 24 h prior to major abdominal oncologic surgery had a higher EF and cardiac index postoperatively [96].

When providing hemodynamic support intraoperatively, care must be taken to avoid excessive afterload, tachycardia, or arrhythmias. In this way, hemodynamic support can be provided without resulting in a greater mismatch between myocardial oxygen supply and demand, decreased diastolic filling time, or arrhythmias which can further exacerbate heart failure. An understanding of the pathophysiology of heart failure and knowledge of the severity of a patient’s disease can help inform clinicians as to the cause of hemodynamic derangements in the perioperative setting. As discussed previously, invasive monitoring serves an important role and can help assess the adequacy of a patient’s contractile function and volume status. These determinations can help clinicians decide whether fluids, vasopressors, or inotropic support are indicated or if a combination of agents should be used. Ultimately, an individualized approach should be taken and more research is currently needed to provide a clear consensus on vasopressor or inotrope choice perioperatively [70,97].

## 6. Postoperative Care

The postoperative period for patients with HF is complicated by the surgical stress response to trauma, increases in cardiac demand, and the need to monitor postoperative fluid dynamics to prevent systemic decline. In particular, in cardiac surgery, HF is associated with increased postoperative complications with circulatory failure and the need for prolonged ventilatory support [98]. Therefore, patients with HF require diligent monitoring to ensure stability during this critical period.

### 6.1. Perioperative Surveillance and Management of Myocardial Injury and Infarction

The 2024 AHA/ACC guidelines highlight myocardial injury after noncardiac surgery as an important disease process that is associated with high postoperative mortality, which correlates with peak elevation of cardiac troponin labs. Although not specific to patients with HF, these patients have pre-existing damage to cardiac function, placing them at higher risk for further injury in the perioperative period. As high-risk patients, consideration should be given to postoperative surveillance for myocardial injury. The AHA/ACC guidelines say it may be reasonable to measure cardiac troponin levels at 24 and 48 h after surgery to identify myocardial injury in patients with known CVD, symptoms of CVD, or age ≥ 65 years with cardiovascular risk factors undergoing elevated-risk noncardiac surgery [38]. The guidelines contrast this with low-risk noncardiac surgery, saying routine postoperative screening is not indicated in the absence of symptoms or signs suggestive of myocardial ischemia or MI.

For patients with HF, screening with cardiac troponin levels, EKG, and other biomarkers may be reasonable for elevated risk surgery or if there is reason to suspect myocardial injury may have occurred. This could include symptoms of myocardial ischemia that are present postoperatively, but it is also important to note that patients with HF may have atypical or absent symptoms, or that in the postoperative period symptoms may not be present due to anesthesia, analgesia, or distracting pain from the operation [99]. Consideration can also be given to surveillance for HF patients if significant hypotension, tachycardia, hypoxia, or other hemodynamic derangements occurred intra- or postoperatively. If myocardial injury is identified in the perioperative period, outpatient follow-up is reasonable for optimization of cardiovascular risk factors [38].

### 6.2. Compensating for Increases in Cardiac Output Demand

The postoperative period is often associated with increased cardiac demand; in the setting of HF, pathological changes can inhibit the ability to sustain sufficient cardiac output. Compensatory mechanisms such as tachycardia can worsen diastolic HF by shortening left ventricular filling times [100]. Low left ventricular volume and benign postoperative arrhythmias can result in hypotension and further exacerbate low cardiac output states [101].

To maintain organ perfusion, postoperative cardiac output may need to be managed with vasopressors as well as inotropic agents [94]. The specific agents used for hemodynamic support intraoperatively can also be used postoperatively and are discussed in detail above. In patients exhibiting signs of malperfusion or those requiring vasopressor or inotropic support during surgery, continued invasive hemodynamic monitoring may be warranted. Anti-arrhythmic medications such as amiodarone and short-acting beta blockers such as esmolol may have benefits in treating postoperative arrhythmias, although beta blockers are not indicated for use in acute decompensated HF [102,103,104].

### 6.3. Monitoring Hemodynamic Changes and Volume Status

During the operative period, large fluid shifts may occur, and careful attention must be paid to volume status in patients with HF who are prone to volume overload and decompensation. Modern anesthetic practices recognize that both intravascular hypovolemia and fluid overload can be harmful and associated with organ dysfunction [105]. Goal-directed fluid therapy aims to deliver perioperative fluids in order to maximize stroke volume and oxygen delivery, often using various monitoring strategies to establish a patient’s volume status, rather than a protocolized approach to fluid administration [106]. However, in patients with HF, classic measurements such as blood pressure, heart rate, and urine output may be unreliable guides for fluid administration. As discussed, invasive monitoring may be required to evaluate fluid status in these patients, and judicious use of intraoperative fluids is recommended. In instances of intraoperative bleeding or hypotension, more aggressive fluid resuscitation may be required, and thus special attention should be paid to monitoring fluid balance in the postoperative period [54].

Patients with HF are prone to pulmonary congestion and edema in hypervolemic states, and respiratory status must be closely monitored in the postoperative period. Standard extubation criteria may be followed for patients with HF; however, non-invasive ventilation or high-flow nasal therapy should be considered for high-risk HF patients immediately following extubation [107,108]. To monitor postoperative ventricular filling and evaluate for fluid overload, bedside echocardiography can be considered [109,110]. Intravenous loop diuretics are the first-line treatment for fluid overload in HF patients, set at 20–40 mg of furosemide for patients not already receiving diuretic therapy, or one to two times the total oral home dose for those who are currently on treatment [111]. Torsemide has significant efficacy in patients with acute decompensated HF [112]. Continuous infusions of high-dose furosemide have also been shown to be effective among patients with severe HF [113].

Improper diuretic usage can lead to development of acute kidney injury (AKI), which is often complicated by pre-existing HF in the postoperative setting [114]. Combination diuretic therapy can induce hyponatremia, hypokalemia, hypomagnesemia, or hypochloremia [111]. As a result, renal function and electrolytes should be routinely monitored. Blood urea nitrogen (BUN) and creatinine are primary indicators for kidney function but may be delayed in reflecting AKI [115]. The glycoprotein neutrophil gelatinase-associated lipocalin (NGAL) is an early, highly sensitive biomarker to AKI that can be trended with BNP to assess the extent of cardiorenal syndromes [115]. Blood pressure trends are not recommended to assess volume status in HF patients, as there are other compensatory mechanisms available to maintain blood pressure in patients with low volume status [115].

### 6.4. Pain Control and Modulation of the Surgical Stress Response

During the surgical stress response, the body responds nonspecifically to surgical trauma through the secretion of catecholamines, cytokines, and pituitary hormones. Initiation of nociceptors leads to widespread activation of the SNS [116]. In addition to generating the release of norepinephrine, sympathetic activation triggers beta-1-adrenergic receptors to release renin, which increases aldosterone secretion and subsequent sodium/water resorption [116]. The normal response to this sympathetic output is an increase in cardiac contractility, peripheral vasoconstriction, and coronary blood flow to maintain perfusion of vital organs [117]. In the setting of HF, both SNS and RAAS are persistently activated to compensate for chronic states of reduced cardiac output, with eventual downregulation in responsiveness to these systems with progressive disease [20]. As a result, these patients must be monitored postoperatively, as a patient’s lack of ability to respond to increased demands may result in ischemia and decompensation.

Current therapeutic guidelines cite pain control and modulation of the hypothalamic–pituitary axis as the mainstays of attenuating the surgical stress response, though evidence of their efficacy in HF is somewhat limited [117]. Opioids can be used to suppress excessive release of corticotrophin and cortisol, though high-dose opioids can induce excessive respiratory depression [117]. Given that this population often is vulnerable for respiratory complications due to pulmonary edema, it is reasonable to consider a multimodal pain regimen to avoid high-dose opioids. Acetaminophen, dexmedetomidine, ketamine, and regional anesthetic techniques are effective pain management strategies that can reduce opioid use in cardiac surgical patients [118]. Non-steroidal anti-inflammatory drugs (NSAIDs) should be used with caution in this population given that HF patients are vulnerable to renal injury [119]. The use of volatile anesthetics, propofol, and high-dose glucocorticoids has been shown to reduce cytokine-induced inflammation during surgery [119]. There is also evidence that benzodiazepines may inhibit cortisol secretion at the hypothalamic level, though their mechanism and significance remain unclear [120]. Despite potential pharmacologic intervention to attenuate the surgical stress response, the overarching evidence shows that the duration and invasiveness of surgical procedures have the largest effect on the magnitude of the surgical stress response [117,120].

## 7. Conclusions

**Figure A1:**
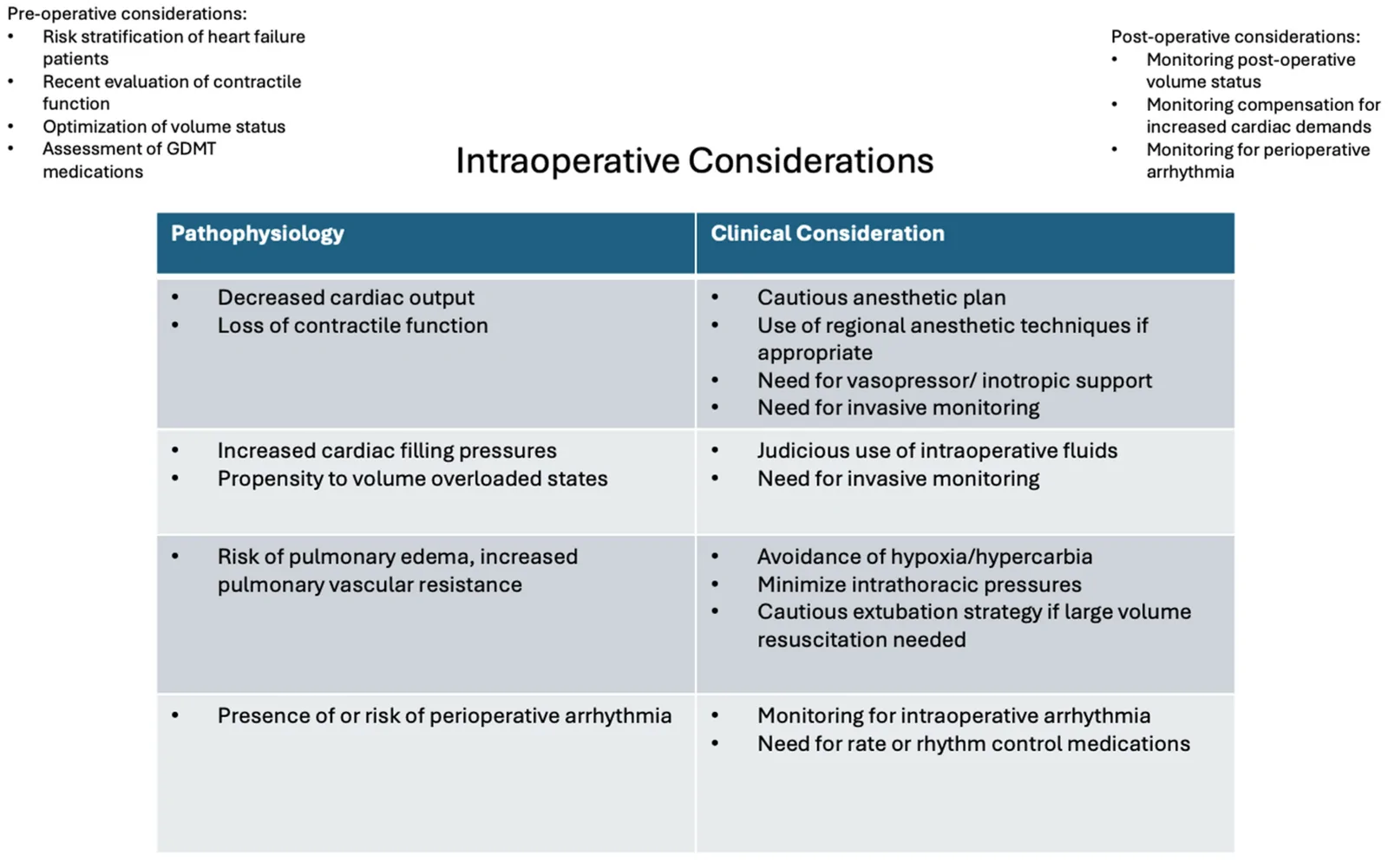
Summary of perioperative considerations for heart failure patients.

The pathophysiology of HF represents a complex network of often self-perpetuating cycles, as decreased cardiac performance leads to mechanisms that initially maintain perfusion but ultimately drive further myocardial injury and disease progression. A thorough understanding of this pathophysiology is essential for anesthetic providers to safely manage patients with this disease. A summary of the perioperative considerations for heart failure patients can be seen in Figure A1. Safe anesthetic management demands an understanding of the hemodynamic derangements of HF, as well as recognition that anesthetic and surgical interventions often well tolerated by healthy individuals may precipitate acute decompensation in HF patients. From the preoperative period with appropriate risk stratification, through postoperative recovery, clinical application of these principles can lead to improved patient care from anesthetic providers.

## Data Availability

No new data were created or analyzed in this study. Data sharing is not applicable to this article.

## Abbreviations

The following abbreviations are used in this manuscript in order of appearance:

HF	Heart failure
IGF	Insulin-like growth factor
SNS	Sympathetic nervous system
RAAS	Renin–angiotensin–aldosterone system
ATII	Angiotensin II
ACE	Angiotensin converting enzyme
SERCA2a	Ca^2+^-ATPase pump
LVEDP	Left ventricular end-diastolic pressure
PAWP	Pulmonary artery wedge pressure
AHA/ACC	American Heart Association/American College of Cardiology
LVEF	Left ventricular ejection fraction
HFrEF	Heart failure with reduced ejection fraction
HFmrEF	Heart failure with mildly reduced ejection fraction
HFpEF	Heart failure with preserved ejection fraction
NYHA	New York Heart Association
ACC/AHA	American College of Cariology/American Heart Association
METs	Metabolic equivalents
RCRI	Revised Cardiac Risk Index
NSQIP	National Surgical Quality Improvement Program
BNP	B-type natriuretic peptide
NT-proBNP	N-terminal pro-BNP
GDMT	Guideline-directed medical therapy
SGLT2	Sodium glucose cotransporter-2
ACEI	Angiotensin converting enzyme inhibitor
ARB	Angiotensin receptor blocker
VGCC	Voltage-gated calcium channel
PIP2	Phosphatidylinositol 4,5 bisphosphate
SVR	Systemic vascular resistance
TIVA	Total intravenous anesthesia
GA	General anesthesia
RA	Regional anesthesia
PPV	Pulse pressure variation
CVP	Central venous pressure
ASA	American Society of Anesthesiologists
TEE	Transesophageal echocardiography
cAMP	Cyclic adenosine monophosphate
AKI	Acute kidney injury
BUN	Blood urea nitrogen
NGAL	Neutrophil gelatinase-associated lipocalin
NSAIDs	Non-steroidal anti-inflammatory drugs
